# A 10-Year Risk Assessment and Primary Prevention Study of Atherosclerotic Cardiovascular Disease Among Adult Patients in Saudi Arabia: A Cross-Sectional Study

**DOI:** 10.3390/medicina61010132

**Published:** 2025-01-15

**Authors:** Abdulhameed Alkhalaf, Qasem Aljabr, Khalid Al Mulla, Duaa Almubarak, Abdul Sattar Khan, Zainab Y. Al Jaziri, Ibrahim Albahrani

**Affiliations:** 1 Family Medicine, Al-Ahsa Health Cluster, Alahsa 31982, Saudi Arabia; duaa.ali.mubarak@hotmail.com (D.A.); z.jaziri97@gmail.com (Z.Y.A.J.); 2 Family Medicine and Geriatric Medicine, Al-Ahsa Health Cluster, Alahsa 31982, Saudi Arabia; dr.qasem.aljabr@gmail.com; 3 Al-Ahsa Health Cluster, Alahsa 31982, Saudi Arabia; kalmulla@moh.gov.sa; 4 King Faisal University, Al Hofuf 31982, Saudi Arabia; amkhan@kfu.edu.sa; 5 Nursing, Al-Ahsa Health Cluster, Alahsa 31982, Saudi Arabia; fedaka110@hotmail.com

**Keywords:** atherosclerotic cardiovascular disease (ASCVD), 10-year ASCVD risk, primary prevention, risk factors, statin therapy, aspirin use, hypertension, diabetes mellitus, cholesterol levels, Saudi Arabia

## Abstract

*Background and Objectives*: Cardiovascular diseases (CVDs), primarily driven by atherosclerosis, are the leading cause of mortality worldwide. In Saudi Arabia, the prevalence of atherosclerotic cardiovascular disease (ASCVD) poses a significant public health challenge. To estimate the 10-year ASCVD risk among adults in Al-Ahsa, Saudi Arabia, and identify prevalent risk factors such as age, gender, diabetes, hypertension, smoking, cholesterol, and preventive medication use. *Materials and Methods*: This cross-sectional study included 58,743 adults aged 35–75 years from the Al-Ahsa Health Cluster. The ASCVD risk was calculated using the ACC/AHA Pooled Cohort Equations. Statistical analysis identified predictors of high ASCVD risk. *Results*: Most participants (76.3%) were at low ASCVD risk (≤10%), 13.3% at borderline to intermediate risk (10–19%), and 10.4% at high risk (≥20%). Smoking, diabetes (39.6%), hypertension (40.8%), and male gender were key predictors of high ASCVD risk. High-density lipoprotein (HDL) was protective, reducing ASCVD risk by 3%. Among high-risk individuals, 29.7% used aspirin, and 58.3% used statins. *Conclusions*: While most adults in Al-Ahsa are at low ASCVD risk, a significant portion remains at elevated risk. Modifiable risk factors, including smoking, diabetes, and hypertension, combined with statin and aspirin adherence, highlight critical areas for targeted interventions to reduce the ASCVD burden in this population.

## 1. Introduction

Cardiovascular diseases (CVDs) are caused by atherosclerosis, which results from plaque buildup in arterial walls that limit blood flow resulting in critical cardiovascular events such as heart attacks and strokes. These diseases are a leading cause of death globally, with an estimated 17.9 million deaths in 2019, accounting for 32% of all global deaths. 85% of these deaths were due to heart attacks and strokes [1]. In 2019, the highest age-standardized rates of coronary heart disease (CHD) incidence and prevalence recorded in the Middle East and North Africa (MENA) region, accounting for 54.7% of cardiovascular disease cases. Iran, Egypt, and Kuwait had the highest rates, while Turkey, Tunisia, and Algeria had lower rates. Saudi Arabia had the highest prevalence of CHD, with 835,249 cases and an age-standardized rate of 4787 per 100,000 [2].

Cardiovascular diseases (CVDs) are a significant health concern in Saudi Arabia, with high prevalence rates and incidence rates. In 2019, they were the leading cause of death and disability. Recent national data indicates that cardiovascular disease (CVD) is responsible for nearly 37% of total deaths in Saudi Arabia. Factors such as hypertension, diabetes, and obesity contribute to the rising prevalence of CVD. Preventive measures and lifestyle modifications are essential to address this growing health challenge [3]. Primary prevention of atherosclerotic cardiovascular disease (ASCVD) involves evaluating risk levels using population-based calculators like the American College of Cardiology/AHA’s pooled cohort equations. Evidence-based guidelines recommend statin and aspirin therapy, as well as aggressive antihypertensive treatment. For intermediate- or uncertain-risk patients, selective utilization of biomarkers, non-modifiable risk factors, social determinants of health, and noninvasive measures can inform treatment decisions [4,5]. As such, the 2018 ACC/AHA guidelines strongly recommend statin therapy for patients with ASCVD, DM, and primary hypercholesterolemia without risk assessment, and for those aged 40–75 after a 10-year ASCVD risk assessment [6].

Many studies have assessed the 10-year risk of ASCVD globally. A study in Karnataka, India, found that 65% of participants were at low risk for cardiovascular disease over the following ten years, whereas 21.2%, 9.7%, and 4.1% were considered to have mild risk, moderate, and high risk levels, respectively. Physical inactivity was prevalent, with a significant associations found between smoking, elevated blood glucose levels, and lower educational status [7]. Another cross-sectional study was conducted in Pakistan found a low risk of atherosclerotic cardiovascular disease (ASCVD) in 69.3% of participants aged 40–79, with a high risk level found in 9.8%. Factors predicting high risk levels included age, male gender, diabetes, smoking, and high cholesterol levels [8]. In 2022, a cross-sectional cohort study was conducted in Iran, which revealed that 75.3% of Shiraz University employee participants aged 25–70 had a low risk of atherosclerotic cardiovascular disease (ASCVD), with higher risks being found for men. Uncontrolled hypertension and diabetes were also identified, suggesting targeted interventions and preventive measures are needed [9].

Locally, in Saudi Arabia, a study conducted through semi-structured interviews with 25 senior Ministry of Health managers found that despite successes in noncommunicable disease (NCD) prevention, inefficient program management and low community awareness were significant challenges. Policies regarding tobacco, sugar-sweetened drinks, and obesity were developed faster than for physical activity [10]. Another cross-sectional study in Riyadh, involving 2997 adults aged 30 to 75, found significant gender differences in chronic diseases and atherosclerotic cardiovascular disease (ASCVD) risk scores. Women were found to have higher prevalence rates of diabetes, hypertension, hypercholesterolemia, and obesity. Men had higher 10-year ASCVD risk and lifetime risk. The study concluded that women aged 50–59 years with multiple risk factors are at a greater risk of developing cardiovascular diseases [11]. However, in Alharja, there was a correlation between high atherosclerotic cardiovascular disease (ASCVD) risk and factors like male gender, age, diabetes, hypertension, smoking, and high cholesterol levels, with males having higher ASCVD scores [12].

This study aimed to estimate the 10-year ASCVD risk among adults in Al-Ahsa, Saudi Arabia, and identify prevalent risk factors, including age, gender, diabetes, hypertension, smoking, and cholesterol. Understanding these factors can guide targeted interventions and improve cardiovascular health outcomes in the region.

## 2. Materials and Methods

### 2.1. Study Design and Population

This cross-sectional study was conducted in Al-Ahsa, Eastern Province, Saudi Arabia. Data were collected from 58,743 adult patients aged 35–75 years attending 64 primary healthcare centers in the Al-Ahsa Health Cluster. Patients with pre-existing cardiovascular diseases were excluded to ensure the applicability of the ASCVD risk calculator, which is designed for primary prevention.

Participants with missing essential data required for ASCVD risk calculation (e.g., cholesterol levels, blood pressure, smoking status) were excluded to prevent bias. For non-critical missing data, multiple imputations were utilized to handle data missing at random, thereby minimizing bias and improving accuracy in ASCVD risk estimation.

Baseline data included age, sex, total cholesterol, HDL, systolic blood pressure, hypertension treatment status, diabetes status, and smoking status. The 10-year ASCVD risk was calculated using the ACC/AHA Pooled Cohort Equations.

### 2.2. Ethical Considerations

Ethical approval was obtained from the Institutional Review Board (IRB) of the Al-Ahsa Health Cluster (IRB Code: H-05-HS-135). Patient confidentiality was maintained by anonymizing data, and findings were reported in aggregate.

### 2.3. Statistical Analysis

Data were analyzed using SPSS version 26. Descriptive statistics summarized demographic characteristics and ASCVD risk factors. Chi-square tests were used for categorical variables, and *t*-tests or ANOVA were used for continuous variables. A stepwise logistic regression model identified predictors of high ASCVD risk, with results presented as adjusted odds ratios (ORs) and 95% confidence intervals (CIs).

## 3. Results

### 3.1. Participant Characteristics

The study included 58,743 participants with a mean age of 50.98 ± 10.03 years. Most participants were female (61.8%), and 12.1% were smokers. Diabetes and hypertension were prevalent in 39.6% and 40.8% of participants, respectively. Among high-risk individuals, 29.7% used aspirin, and 58.3% used statins (Table 1).

### 3.2. ASCVD Risk Distribution

**Figure 1:**
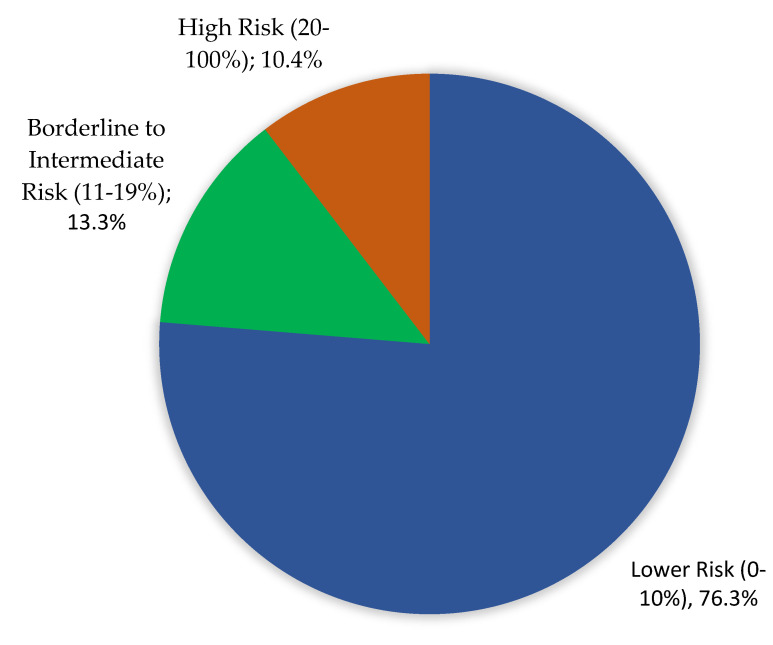
Risk prediction based on 10-year study of Atherosclerotic Cardiovascular Disease among Adults in Al-Ahsa, Saudi Arabia.

The 10-year ASCVD risk was classified as low (≤10%) in 76.3% of participants, borderline to intermediate (10–19%) in 13.3%, and high (≥20%) in 10.4% (Figure 1).

**Figure 2:**
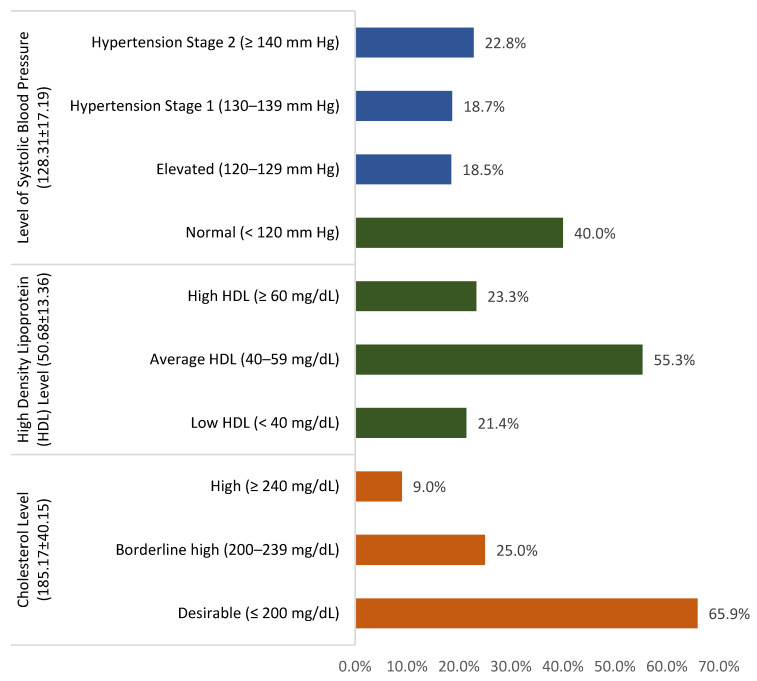
Risk factors for Atherosclerotic Cardiovascular Disease among Adults in Al-Ahsa, Saudi Arabia.

Figure 2 illustrates the critical risk factors for Atherosclerotic Cardiovascular Disease (ASCVD) among adults in Al-Ahsa. Elevated systolic blood pressure (SBP) was observed in 18.5% of participants, while 41.5% of participants experiencing hypertension varied between stage 1 and 2 (18.7%, 22.8%), respectively. More than half of participants (55.3%) showed average levels of HDL cholesterol. Additionally, 23.3% had high HDL levels; on the other hand, low levels were reported in 21.4%. Cholesterol levels were borderline high in 25% of participants and high in 9%.

Table 2 provides the factors associated with risk Prediction 10 years of Atherosclerotic Cardiovascular Disease among Adults in Al-Ahsa, Saudi Arabia. A significant difference across various demographics and health conditions was observed. Also, a significant positive correlation between age and high ASCVD risk was noted, with 86% of adults at risk being aged 71–75, compared to only 0.1% of those aged 35–39 (*p* = 0.001). Gender differences were also significant, as males exhibited a notably higher 10-year risk, at 25.4%, while females had a risk of only 8.3%. This underscores gender as an important risk factor in the progression of ASCVD (*p* = 0.001).

A significant risk being incurred by smoking is noticeable, with 25.4% of smokers categorized as high risk compared to only 8.3% of non-smokers. In comparison to the 23.9% of diabetic participants categorized as high risk, only 1.5% of those non-diabetic have this categorization. This further reinforces diabetes as a significant predictor of risk for atherosclerotic cardiovascular disease (ASCVD) (*p* = 0.001).

Regarding cholesterol level as a risk factor, 12% of participants with high cholesterol level were categorized as high risk in comparison to 10.9% with desirable cholesterol levels. This indicates a need for effective cholesterol management (*p* = 0.001). Similarly, HDL level plays a major role, with 18.2% of individuals with low HDL being classified as high risk for ASCVD, while only 5.2% of those with high HDL fell into the high-risk category, which raises questions regarding the impact of HDL as a protective factor in ASCVD prevention.

Regarding the impact of Blood pressure, 24.7% of adults in hypertension stage 2 were classified as high risk, which is significantly higher than the 3.1% prevalence of high risk levels among individuals with normal blood pressure levels.

With respect to the labeled data above, geographic variation within Al-Ahsa indicated that residents of the middle zone had the highest risk, at 11.3%, suggesting that lifestyle or environmental factors may influence ASCVD risk. According to these data, there is a need for tailored interventions that focus on age, gender, smoking, diabetes, cholesterol, HDL levels, blood pressure, and geographic residence to reduce ASCVD risk among the population of Al-Ahsa effectively.

### 3.3. Predictors of High ASCVD Risk

Stepwise logistic regression identified smoking (OR: 35.1; 95% CI: 33.2–39.4), diabetes mellitus (OR: 18.7; 95% CI: 16.8–20.4), hypertension (OR: 4.54; 95% CI: 3.7–5.1), and male gender (OR: 3.58; 95% CI: 2.7–5.9) as significant predictors of high ASCVD risk. Higher HDL levels were protective, reducing ASCVD risk by 3% (Table 3).

These findings emphasize the need for targeted interventions focused on smoking cessation, diabetes management, hypertension control, and gender-specific prevention strategies to mitigate ASCVD risk in Al-Ahsa.

### 3.4. Aspirin and Statin Use

Table 4 demonstrates the use of aspirin (ASA) and statins among adults in Al-Ahsa, categorized by different ASCVD risk levels. Ideally, all individuals classified as having borderline, intermediate, or high ASCVD risk should be using ASA and statins; however, the data reveal significant gaps in adherence to these guidelines.

In the high-risk group, only 29.7% reported using ASA, while more than 70% did not. Regarding those with borderline to intermediate risk, approximately 80% did not use ASA.

Regarding statin usage, in the high-risk, borderline-to-intermediate-risk, and low-risk groups, only 58.3%, 49.2%, and 15.7% were on statin, respectively.

Based on evidence-based guidelines for reducing the incidence of ASCVD by using ASA and statin, urgent improvements in using them are warranted among borderline-to-intermediate and high-risk groups. Enhancing adherence to preventive therapy in these populations could significantly lower ASCVD risk and promote long-term cardiovascular health in Al-Ahsa.

Table 5 highlights the distribution of statin use among adults in Al-Ahsa by 10-year ASCVD risk across various geographic zones. Sarting with the Middle zone, in high-risk individuals, only 59.7% were on statin therapy while 40.3% were not. Among the borderline-to-intermediate risk group, using statin or not seems to be half-by-half, with 51.9% and 48.1%, respectively. In the low-risk category, only 16.3% received statins, compared to 83.7%, who did not (*p* = 0.001), indicating a selective approach based on ASCVD risk level.

In the Northern zone, in high-risk individuals, only 55.2% were on statin therapy while 44.8% were not. Regarding the borderline-to-intermediate risk group, use of statin or lack thereof seems to be half-by-half, with 46.8% and 53.2%, respectively. In the low-risk group, only 14.7% were on statins, while 85.3% did not use them (*p* = 0.001).

In the Eastern zone, in high-risk individuals, 63.8% were on statin therapy and only 36.2% were not. Regarding the borderline to intermediate risk group, use of statin or lack thereof seems to be half-by-half, with 51.2%, and 48.8%, respectively. In the low-risk category, 17.2% were on statins, and 82.8% were not receiving therapy (*p* = 0.001).

In the Southern zone, for high-risk individuals, only 50.4% were on statin therapy and 49.6% were not. Within the borderline-to-intermediate risk group, 44.2% were on statins, while 55.8% did not receive statin therapy. In the low-risk group, 14.3% used statins, while 85.7% did not (*p* = 0.001).

For the NA (not classified) zone, statin use occurred in 18.7% of all cases. Among high-risk individuals, use of statin or lack thereof seems to be half-by-half, at 51.0% and 49%, respectively. Similarly, in the borderline-to-intermediate risk group, 48.8% were on statins, while 51.2% were not. In the low-risk category, 12.4% used statins, while 87.6% did not (*p* = 0.001).

Table 6 outlines the distribution of aspirin (ASA) use among adults in Al-Ahsa, segmented by 10-year ASCVD risk levels and geographic zones. Starting with the middle zone, in high-risk individuals, only 29.8% were taking ASA, whereas 70.2% were not. Additionally, in the borderline-to-intermediate risk group, 20.2% of individuals used ASA, while 79.8% did not. In contrast, among those in the low-risk group, only 3.8% were on ASA therapy, with 96.2% not using it (*p* = 0.001).

In the Northern zone, high-risk ASA usage was reported to be 27.0%, meaning 73.0% of high-risk individuals remained untreated. Among borderline-to-intermediate risk individuals, ASA use was 17.9%, with 82.1% being untreated. Low-risk individuals showed an ASA usage rate of 2.8%, with 97.2% not using ASA (*p* = 0.001).

Compared to the Eastern zone, ASA usage was 35.5% in the high-risk group, 22.3% in the borderline to intermediate risk group, and only 3.9% for participants in the low-risk group.

The results for the Southern zone showed that ASA usage applied to only 23.2% in the high-risk group, 13.4% in the borderline-to-intermediate risk group, and 2.8% of participants in the low-risk group.

In the NA (not classified) zone, ASA use was 5.1% overall. Within the high-risk group, 20.4% were on ASA therapy, while 79.6% remained untreated. In the borderline to intermediate group, 14.4% used ASA, with 85.6% not using the medication. Low-risk individuals had an ASA usage rate of 2.6%, with 97.4% not using ASA (*p* = 0.001).

## 4. Discussion

The primary finding of this study is that most adults in Al-Ahsa, Saudi Arabia, exhibit a low 10-year ASCVD risk, with 76.3% being classified as low risk. However, a significant proportion of the population—10.4%—were identified as high risk, and an additional 13.3% fell into the borderline-to-intermediate risk category. These findings highlight the dual challenge of maintaining low-risk status in the majority while addressing the elevated risk in a notable subset of the population. This result is consistent with previous studies in Saudi Arabia, such as Almulhim et al., who reported a similarly high prevalence of ASCVD risk among older adults and males in the region, emphasizing the influence of non-modifiable risk factors like age and gender [13]. Saeed et al. also observed a higher ASCVD risk in males and older populations, findings that are further supported by regional studies conducted in Al-Madinah by Qasem et al. and in other Middle Eastern populations by Abril-López et al. [12,14,15]. These consistent observations across studies underscore the need for targeted preventive strategies, particularly for older individuals and males, who represent the highest-risk groups.

In terms of modifiable risk factors, the study identified smoking, diabetes mellitus (DM), hypertension, and low HDL cholesterol as significant contributors to elevated ASCVD risk. The high prevalence of these factors in Al-Ahsa adults—39.6% with DM and 40.8% with hypertension—aligns with findings from Alhabib et al., who reported similarly high rates of obesity, dyslipidemia, hypertension, and diabetes in Saudi Arabia [16]. Compared with neighboring countries, such as Lebanon (29.8% DM prevalence) and Bahrain (36.9% DM prevalence), the rates in Al-Ahsa are notably higher, reflecting the growing burden of metabolic and lifestyle-related risk factors in Saudi Arabia [17,18]. These findings emphasize the critical need for public health interventions targeting smoking cessation, better glycemic control, and improved management of hypertension and dyslipidemia. Nakhaie et al. similarly highlighted the importance of addressing these modifiable risk factors, particularly in populations with high workplace stress and sedentary lifestyles [19].

Another key finding of this study is the low adherence to preventive medications among high-risk individuals. Only 29.7% of high-risk adults were on aspirin, and 58.3% were on statins, despite clear evidence supporting their benefits in reducing ASCVD risk. Statin use was highest among high-risk individuals, yet a significant proportion of this group remained untreated. Geographic differences in statin use were also observed, with the Eastern zone showing the highest adherence (63.8%) and the Southern zone the lowest (50.4%). These disparities may reflect differences in healthcare access, socioeconomic factors, or awareness of preventive guidelines. Similar trends have been observed in broader studies of medication adherence, which highlight the role of patient education, affordability, and provider prescribing practices in influencing adherence rates. Addressing these barriers is essential to improving the uptake of evidence-based therapies and reducing the overall burden of ASCVD.

Aspirin use in primary prevention remains a complex and debated topic due to the bleeding risks associated with its use. In line with updated guidelines from the AHA/ACC and the European Society of Cardiology, which recommend limiting aspirin use in primary prevention to high-risk individuals with low bleeding risk, our study found low aspirin use (8.2%) overall [20,21,22]. This cautious approach is supported by findings from recent trials such as ASPREE, ARRIVE, and ASCEND, which demonstrated that the benefits of aspirin in primary prevention are often outweighed by bleeding risks, particularly when statins are concurrently used to reduce ASCVD risk [23,24,25,26,27,28]. Our data reflect this trend, with aspirin use significantly lower than statin use among high-risk groups. Regional variability in aspirin use also suggests differences in prescribing practices or patient preferences, warranting further investigation to better understand these patterns and optimize the use of aspirin in appropriate cases.

### 4.1. Clinical Implications

The findings of this study underscore the critical role of non-modifiable (age and gender) and modifiable (smoking, diabetes mellitus, hypertension, and low HDL) factors in ASCVD risk stratification within the Al-Ahsa region. To address these risks, public health initiatives should prioritize strategies to reduce modifiable risk factors, including implementing structured smoking cessation programs, comprehensive diabetes management, and hypertension control protocols, as well as encouraging lifestyle modifications to improve HDL levels through diet and regular physical activity.

Furthermore, adherence to evidence-based therapies such as statins and aspirin is essential for high-risk groups, guided by ACC/AHA recommendations for primary prevention. Our study revealed lower-than-expected adherence rates in specific zones within Al-Ahsa, highlighting the need for improved patient and healthcare provider education on preventive medication’s long-term benefits and safety. Providing tailored information on these therapies could significantly improve adherence, ultimately reducing ASCVD risk across the population.

An interdisciplinary approach involving dietitians, smoking cessation counselors, and other specialists would further support comprehensive risk management. Measuring the success of these initiatives through specific metrics—such as reductions in smoking rates, improvements in blood pressure, and increases in HDL levels—will provide actionable insights into the efficacy of these strategies over time.

Through focused, regionally tailored efforts, the ASCVD burden in Al-Ahsa can be more effectively managed, leading to improved patient outcomes and reduced long-term cardiovascular risk.

### 4.2. Study Limitations

Despite its strengths, this study has several limitations. First, the reliance on a pre-existing database limited the availability of critical data, such as family history, detailed lifestyle factors (e.g., smoking intensity or diet), and prior cardiovascular events. This lack of granularity may have influenced the accuracy of ASCVD risk estimations and prevented a clear distinction between primary and secondary prevention contexts. Second, the exclusion of non-Saudis and individuals with missing data introduces the potential for selection bias, limiting the generalizability of the findings to the broader Al-Ahsa population. Third, the cross-sectional design precludes the ability to observe changes in ASCVD risk factors or medication adherence over time, which would provide a more dynamic understanding of risk progression and the long-term impact of preventive strategies.

Future studies should address these limitations by incorporating longitudinal designs that monitor changes in ASCVD risk factors and adherence to preventive therapies over time. Additionally, direct patient interviews or assessments could provide more detailed clinical and lifestyle information, including the specific purposes of prescribed medications. Investigating the cultural, economic, and healthcare system factors that influence ASCVD risk and prevention in Al-Ahsa could also offer valuable insights for designing targeted interventions. Furthermore, qualitative studies exploring the perspectives of both patients and healthcare providers could help identify barriers to adherence and inform the development of tailored strategies to improve medication use and overall cardiovascular health.

## 5. Conclusions

The study highlights that while most adults in Al-Ahsa are at low ASCVD risk, a significant portion remains at elevated risk due to modifiable factors such as smoking, diabetes, and hypertension. Efforts to enhance adherence to preventive therapies and address regional disparities in care are critical to reducing the ASCVD burden in this population.

## Figures and Tables

**Table 1:** Basic Participants Characteristics (n = 58,743).

Variables (Mean ± SD *)	Categories	Frequency	Percentage
Zone	Middle	16,545	28.2
	Northern	15,179	25.8
	Eastern	16,982	28.9
	Southern	8711	14.8
	NA	1326	2.3
Age (50.98 ± 10.034)	35–39 Years	7971	13.6
	40–45 Years	12,781	21.8
	46–50 Years	9765	16.6
	51–55 Years	8703	14.8
	56–60 Years	7592	12.9
	61–65 Years	6585	11.2
	66–70 Years	3464	5.9
	71–75 Years	1883	3.2
Sex	Male	22,428	38.2
	Female	36,315	61.8
Smoking	Yes	7138	12.1
	No	51,624	87.9
Diabetic	Yes	23,282	39.6
	No	35,480	60.4
Hypertensive	Yes	23,962	40.8
	No	34,800	59.2
Using Aspirin	Yes	4822	8.2
	No	53,940	91.8
Taking Statin	Yes	14,455	24.6
	No	44,307	75.4

* Standard Deviation.

**Table 2:** Factors associated with risk: prediction based on 10-year study of atherosclerotic cardiovascular disease among adults in Al-Ahsa, Saudi Arabia.

Factors	ASCVD—Risk in Percentage						*p*-Value
	Low-Risk		Borderline-to-Intermediate Risk		High Risk		
	No	%	No	%	No	%	
Age in Years							0.001 *
35–39	7914	99.3%	46	0.6%	11	0.1%	
40–45	12,469	97.6%	261	2.0%	50	0.4%	
46–50	9169	93.9%	487	5.0%	109	1.1%	
51–55	7444	85.5%	994	11.4%	265	3.0%	
56–60	5054	66.6%	1839	24.2%	699	9.2%	
61–65	2414	36.7%	2640	40.1%	1531	23.2%	
66–70	334	9.6%	1319	38.1%	1811	52.3%	
71–75	31	1.6%	233	12.4%	1619	86.0%	
Sex							0.001 *
Male	13,687	61.0%	4483	20.0%	4258	19.0%	
Female	31,142	85.8%	3336	9.2%	1837	5.1%	
Smoking							0.001 *
Yes	3518	49.3%	1808	25.3%	1811	25.4%	
No	41,311	80.1%	6011	11.6%	4284	8.3%	
Diabetes mellitus							0.001 *
Yes	11,926	51.2%	5774	24.8%	5574	23.9%	
No	32,903	92.8%	2045	5.8%	521	1.5%	
Cholesterol Level							0.043 *
0.00–200.00—Desirable	29,374	75.8%	5138	13.3%	4223	10.9%	
200.01–239.00 Borderline	11,629	79.1%	1835	12.5%	1235	8.4%	
239.01–500—High	3826	72.1%	846	15.9%	637	12.0%	
HDL Level							0.001 *
≤40—Low	7853	62.5%	2417	19.2%	2287	18.2%	
41–59 Average	25,097	77.3%	4283	13.2%	3098	9.5%	
≥60 High	11,879	86.7%	1119	8.2%	710	5.2%	
Blood Pressure							0.001 *
Normal Blood Pressure (≤120)	21,102	89.9%	1645	7.0%	727	3.1%	
Elevated (121–129)	8800	81.0%	1294	11.9%	776	7.1%	
Hypertension Stage 1 (130–139)	7798	71.0%	1911	17.4%	1272	11.6%	
Hypertension Stage 2 (≥140)	7129	53.1%	2969	22.1%	3320	24.7%	
Zone							0.047 *
Middle	12,302	74.4%	2370	14.3%	1873	11.3%	
Northern	11,994	79.0%	1796	11.8%	1389	9.2%	
Eastern	12,871	75.8%	2310	13.6%	1801	10.6%	
Southern	6559	75.3%	1218	14.0%	934	10.7%	

*p*: Pearson X^2^ test. * *p* < 0.05 (significant).

**Table 3:** Stepwise logistic regression model identifying predictors of high ASCVD 10-year risk.

Risk Factors	B	S.E.	*p*-Value	ORA	95% CI	
					LL	UL
HDL (high level)	−0.045	0.044	0.005 *	0.97	0.85	0.99
Smoking	3.559	0.068	0.001 *	35.1	33.2	39.4
Hypertension	1.514	0.046	0.001 *	4.54	3.7	5.1
Diabetes Mellitus	2.927	0.053	0.001 *	18.7	16.8	20.4
Age in years	0.26	0.002	0.001 *	1.29	1.28	1.31
Male gender	3.478	0.057	0.006 *	3.58	2.7	5.9

B: regression co-efficient. ORA: adjusted odds ratio. CI: confidence interval. * *p* < 0.05 (significant).

**Table 4:** Distribution of aspirin and statin intake by adults, based on 10-year risk study of ASCVD.

Medications		ASCVD 10-Year Risk			Total	*p* Value
		Lower Risk	Borderline-to-Intermediate Risk	High Risk		
ASA						0.001 *
Yes	No	1516	1496	1809	4821	
	% within ASCVD—Risk	3.4%	19.1%	29.7%	8.2%	
No	No	43,313	6323	4286	53,922	
	% within ASCVD—Risk	96.6%	80.9%	70.3%	91.8%	
Statin						0.001 *
Yes	No	7045	3850	3555	14,450	
	% within ASCVD—Risk	15.7%	49.2%	58.3%	24.6%	
No	No	37,784	3969	2540	44,293	
	% within ASCVD—Risk	84.3%	50.8%	41.7%	75.4%	

*p*: Pearson X^2^ test. * *p* < 0.05 (significant).

**Table 5:** The distribution of statin and statin intake by adults in 10-year risk study of ASCVD for different zones.

Zone	Statin	Total	ASCVD—Risk in Percentage						*p*-Value
			Lower Risk		Borderline-to-Intermediate Risk		High Risk		
			No	%	No	%	No	%	
Middle	Yes	4347 (26.3%)	2000	16.3%	1229	51.9%	1118	59.7%	0.001 *
	No	12,198 (73.7%)	10,302	83.7%	1141	48.1%	755	40.3%	
Northern	Yes	3369 (22.2%)	1762	14.7%	840	46.8%	767	55.2%	0.001 *
	No	11,810 (77.8%)	10,232	85.3%	956	53.2%	622	44.8%	
Eastern	Yes	4542 (26.7%)	2211	17.2%	1182	51.2%	1149	63.8%	0.001 *
	No	12,440 (73.3%)	10,660	82.8%	1128	48.8%	652	36.2%	
Southern	Yes	1944 (22.3%)	935	14.3%	538	44.2%	471	50.4%	0.001 *
	No	6767 (77.7%)	5624	85.7%	680	55.8%	463	49.6%	
NA	Yes	248 (18.7%)	137	12.4%	61	48.8%	50	51.0%	0.001 *
	No	1087 (81.3%)	966	87.6%	64	51.2%	48	49.0%	

*p*: Pearson X^2^ test. * *p* < 0.05 (significant).

**Table 6:** The distribution of aspirin and statin intake by adults based on 10-year risk study of ASCVD in different zones.

Zone	ASA	Total		ASCVD—Risk in Percentage						*p*-Value
				Lower Risk		Borderline-to-Intermediate Risk		High Risk		
		No	%	No	%	No	%	No	%	
Middle	Yes	1508	9.1%	471	3.8%	479	20.2%	558	29.8%	0.001 *
	No	15,037	90.9%	11,831	96.2%	1891	79.8%	1315	70.2%	
Northern	Yes	1032	6.8%	336	2.8%	321	17.9%	375	27.0%	0.001 *
	No	14,147	93.2%	11,658	97.2%	1475	82.1%	1014	73.0%	
Eastern	Yes	1653	9.7%	499	3.9%	515	22.3%	639	35.5%	0.001 *
	No	15,329	90.3%	12,372	96.1%	1795	77.7%	1162	64.5%	
Southern	Yes	561	6.4%	181	2.8%	163	13.4%	217	23.2%	0.001 *
	No	8150	93.6%	6378	97.2%	1055	86.6%	717	76.8%	
NA	Yes	67	5.1%	29	2.6%	18	14.4%	20	20.4%	0.001 *
	No	1259	94.9%	1074	97.4%	107	85.6%	78	79.6%	

*p*: Pearson X^2^ test. * *p* < 0.05 (significant).

## Data Availability

The original contributions presented in this study are included in the article. Further inquiries can be directed to the corresponding author.
